# Mesenchymal non-meningothelial tumors of the central nervous system: a literature review and diagnostic update of novelties and emerging entities

**DOI:** 10.1186/s40478-023-01522-z

**Published:** 2023-02-03

**Authors:** Arnault Tauziède-Espariat, Lauren Hasty, Alice Métais, Pascale Varlet

**Affiliations:** 1 Department of Neuropathology, Sainte-Anne Hospital, 1, rue Cabanis, 75014 Paris, France; 2 Inserm, UMR 1266, IMA-Brain, Institut de Psychiatrie et Neurosciences de Paris, Paris, France

**Keywords:** Mesenchymal, DNA-methylation profiling, Classification, Central nervous system

## Abstract

The fifth edition of the World Health Organization Classification of Tumors of the Central Nervous System (CNS) now includes mesenchymal tumors that occur uniquely or frequently in the CNS. Moreover, this version has aligned the terminology of mesenchymal tumors with their soft tissue counterparts. New tumor types have been added, such as the “intracranial mesenchymal tumor, FET-CREB fusion-positive”, the “*CIC*-rearranged sarcoma”, and the “Primary intracranial sarcoma, *DICER1*-mutant”. Other entities (such as rhabdomyosarcoma) have remained in the current WHO classification because these tumor types may present specificities in the CNS as compared to their soft tissue counterparts. Based on an extensive literature review, herein, we will discuss these newly recognized entities in terms of clinical observation, radiology, histopathology, genetics and outcome, and consider strategies for an accurate diagnosis. In light of this literature analysis, we will also introduce some potentially novel tumor types.

## Introduction

**Fig. 1:**
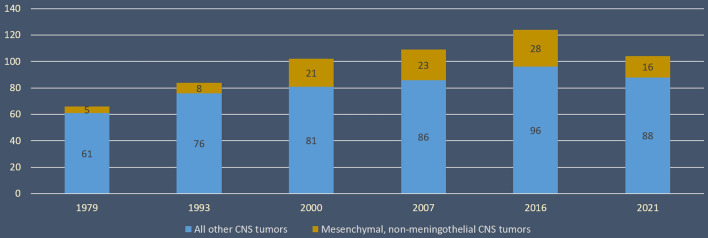
Evolution of the proportion of the number of mesenchymal tumors in the World Health Organization Classification of Central Nervous System according to the versions. *CNS* central nervous system

**Fig. 2:**
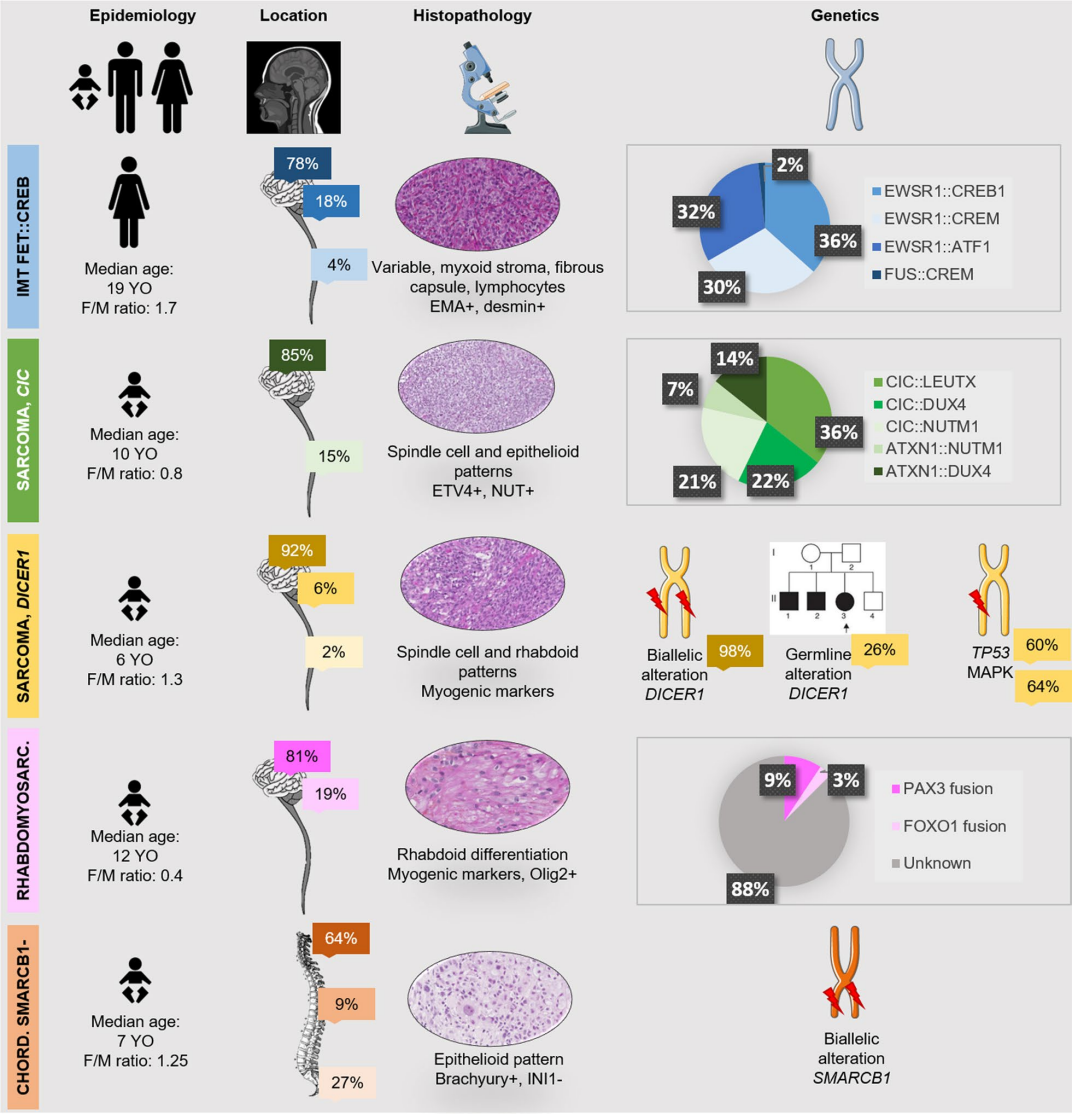
Summary of clinical, histopathological and molecular findings of the new mesenchymal tumor types of the World Health Organization and novelties of previously recognized entities. *Chord*. chordoma, *F* female, *IMT* intracranial mesenchymal tumor, *M* male, *Rhabdomyosarc*. rhabdomyosarcoma, *YO* years old

Mesenchymal non-meningothelial tumors have always been included in the World Health Organization Classification of Tumors of the Central Nervous System (WHO CNS5). The WHO CNS5 is based on the cell of origin (fibroblastic, endothelial, muscular, cartilaginous, notochoral or undetermined) and the advances of genetic and epigenetic data. The WHO CNS5 considerably modified the section on mesenchymal, non-meningothelial tumors. Indeed, this new version covers only tumor types that have special histopathological or molecular features, and occur uniquely in the CNS, or because they are relatively common in the CNS as compared to other tissues. On the one hand, many tumor types, which are common in soft tissue and only exceptionally found in the CNS (such as lipoma, angiolipoma, hibernoma, liposarcoma, osteoma, osteosarcoma, osteochondroma, epithelioid haemangioendothelioma, angiosarcoma, leiomyoma, leiomyosarcoma, fibrosarcoma, desmoid-type fibromatosis, myofibroblastoma, and inflammatory myofibroblastic tumor), and have been present since 2000, have been removed from the current classification. On the other hand, new histomolecular entities have been added, like the intracranial mesenchymal tumor, FET::CREB fusion–positive, *CIC*-rearranged sarcoma, and primary intracranial sarcoma, *DICER1*-mutant. Despite this increase in histomolecular deciphering, and because of this modified nosological organization within the classification, the proportion of mesenchymal, non-meningothelial tumors in the spectrum of all CNS tumors has artificially decreased (Fig. 1). Reflecting this tendency, a specific paragraph dedicated to mesenchymal tumors was written in the review presenting the novel entities of the WHO CNS5 [1]. Based on an extensive literature review, the aims of this discussion are to present the clinical, radiological, histopathological and molecular findings of the newly introduced mesenchymal tumor types found in the classification and novelties of previously recognized entities (Fig. 2). The last part of this review concerns potentially novel subgroups described in the recent literature.

## Newly introduced mesenchymal tumors in the last WHO classification

### Intracranial mesenchymal tumor (IMT), FET::CREB fusion-positive

#### Clinical and radiological characteristics

**Fig. 3:**
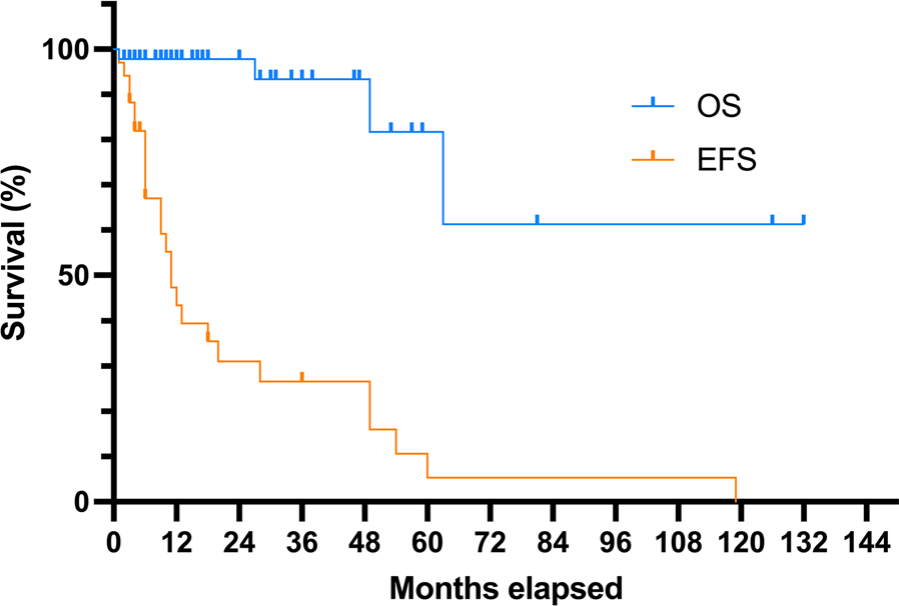
Results of the meta-analysis and prognostic data of IMT, FET::CREB-fused. Results of the meta-analysis including 72 IMT, FET::CREB-fused. Kaplan–Meier estimates of overall survival (OS) and event free survival (PFS). The median OS is not reached. The median EFS is 11 months. *IMT* intracranial mesenchymal tumor

IMT, FET::CREB fusion-positive tumors develop predominantly in supratentorial sites (78% of reported cases) [2–29] but can also be located in infratentorial sites (17% of reported cases) [2, 23, 25, 30–33] and in the spine (5% of reported cases) [11, 23, 34]. They are extra-axial, attached to the meninges or dura [23, 25], or are intraventricular [2, 6, 15, 23, 25, 26, 28]. Presenting symptoms depend on the tumor’s location. Most cases occur in children or young adults with a median age of 19 at diagnosis (ranging from 4 to 79 years) [2–36]. They predominantly affect females (representing 62% of reported cases) [2–36]. In 21% of reported cases, a previous history of cancer (lymphoma or carcinoma) has been reported [2–36]. Radiologically, tumors are hypointense on T1-weighted sequences and are variably intense on T2-weighted images [25]. Lesions present an enhanced tissular portion, and lobulated contours with frequent cystic components [25]. Contrary to meningiomas, a dural tail is rarely observed [25]. The clinical behavior of these tumors seems to be heterogeneous: a subset of cases have aggressive outcomes with local recurrences [6, 13, 21–23, 25, 28, 29, 33], metastases [11, 18, 23, 36] and 8% of reported patients have died from the disease [18, 23, 36] (Fig. 3).

#### Histopathology and cellular origin

**Fig. 4:**
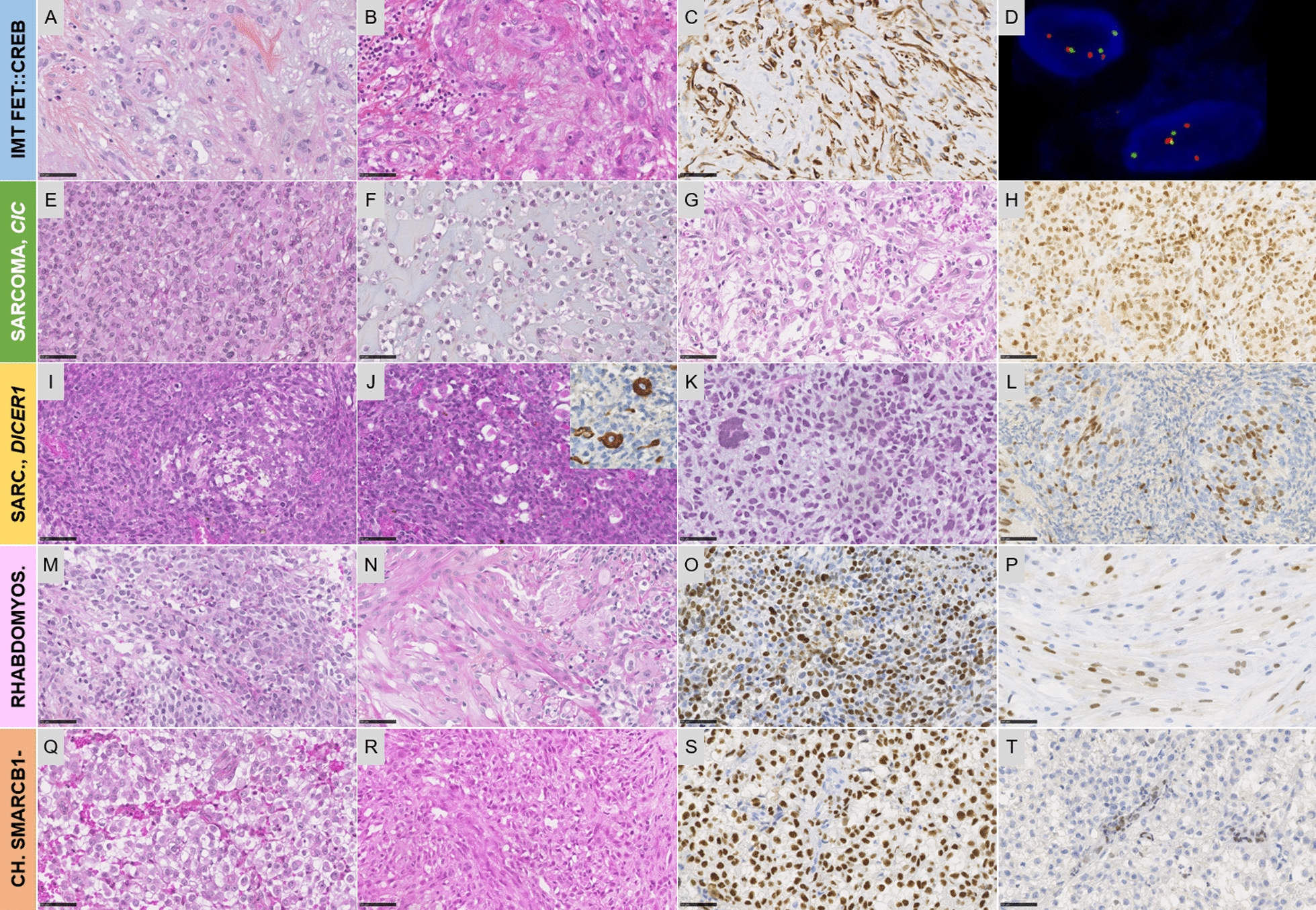
Histopathological and molecular findings of the new mesenchymal tumor types of the World Health Organization and novelties of previously recognized entities. **A** Epithelioid cells in a myxoid stroma with amianthoid fibers and scattered lymphocytes (HPS, magnification × 400). **B** Spindle and epitheliod cells (HPS, magnification × 400). **C** Desmin immunoexpression (magnification × 400). **D** *EWSR1* rearrangement using FISH analysis showing split signals (3’EWSR1: red signals; 5’EWSR1: green signals). **E** Sheets of epithelioid cells (HPS, magnification × 400). **F** Myxoid change (HPS, magnification × 400). **G** Spindle cells and glial differentiation (HPS, magnification × 400). **H** Diffuse expression of ETV4 (magnification × 400). **I** Spindle cell neoplasm with fascicular pattern (HPS, magnification × 400). **J** Myogenic differentiation (HPS, magnification × 400, and insert desmin immunoexpression, magnification × 400). **K** Pleomorphic cells (HPS, magnification × 400). **L** Expression of myogenin (magnification × 400). **M** Sheets of poorly differentiated cells (HPS, magnification × 400). **N** Myogenic differentiation (HPS, magnification × 400). **O** Olig2 immunoexpression described in cases with *PAX3* fusions (magnification × 400). **P** Myogenin immunoexpression (magnification × 400). **Q** Epithelioid cells (HPS, magnification × 400). **R** Spindle cells (HPS, magnification × 400). **S** Brachyury immunoexpression (magnification × 400). **T** Loss of INI1 expression in tumor cells (magnification × 400). Black scale bars represent 50 μm. *Ch*. chordoma, *FISH* fluorescent in situ hybridization, *HPS* hematoxylin phloxin saffron, *IMT* intracranial mesenchymal tumor, *Rhabdomyos*. rhabdomyosarcoma, *sarc* sarcoma

These tumors are multinodular and well-circumscribed from the brain parenchyma, frequently surrounded by a fibrous pseudocapsule. Dense lymphoplasmacytic cuffing at the tumor periphery or intratumoral lymphoplasmacytic infiltrates are typically observed. The stroma may be collagenous (with amianthoid fibers), myxoid or mucin-poor (Fig. 4A, B) [22, 23, 25]. The cellular density is variable and different patterns have been reported (from syncytial or sheet-like growth to reticular cord-like structures) [22, 23, 25]. Tumor cell morphology varies from epithelioid/rhabdoid cells to stellate/spindle cells or monotonous round cells (Fig. 4A, B) [22, 23, 25]. Mitotic activity is generally low but cases with high proliferative indexes have been described at diagnosis or during recurrence [23, 25, 36]. Morphological features reminiscent to meningiomas (such as intranuclear cytoplasmic inclusions and whorls) have been noted [22, 23, 25]. Calcifications (but no psammoma bodies), osseous metaplasia and a pseudochondroid matrix may be exceptionally observed [25]. Using immunohistochemistry, CD99, CD68, desmin and EMA are frequently expressed in various ways (focal to diffuse) (Fig. 4C) [22, 23, 25]. SSTR2a is not stained or only focally on tumor cells [23, 25]. Ultrastructural analyses revealed, in one study, the presence of junction-type desmosomes, *zonula occludens, zonula adherens*, suggesting an arachnoidal origin for tumor cells [25]. These results and DNA-methylation profiling analyses have demonstrated that IMT, FET::CREB fusion-positive are distinct from angiomatoid fibrous histiocytomas of soft tissue [23, 25]. Therefore, the proposed terminology of the WHO classification is provisional and has to be improved.

#### Molecular characteristics

The molecular hallmark of IMT is represented by a fusion of a FET gene with genes from the CREB (cAMP response element) family genes. In the FET family genes, fusions reported in IMT encompass mostly *EWSR1* (97% of reported cases) [2–24, 26–35] while *FUS* was only reported in one case [23] and no fusion implicating *TAF15* gene has been reported to date. These data make the fluorescence in situ hybridization for *EWSR1* a potentially useful diagnostic tool when histology is in line with a diagnosis of IMT (Fig. 4D). From the CREB family genes, *CREB1*, *CREM* and *ATF1* are equally distributed (representing 31, 36 and 33% of partner genes respectively) in fusions encountered in IMT [2–36], contrary to other extra-CNS tumors with FET::CREB fusion (like angiomatoid fibrous histiocytomas of the soft tissue having more than 80% of reported cases described with an *EWSR1::CREB1* fusion). Whereas no methylation class exists in the DKFZ classifier of CNS tumors (v12.5), two recent studies have shown that IMT, FET::CREB fusion-positive are characterized by a distinct epigenetic profile from other tumors of the CNS, but do not represent an homogeneous methylation class [23, 25]. One of them suggests that they are subdivided into two methylation groups (A and B) [23]. Further studies are needed to clearly delineate the epigenetic boundaries of IMT and their clinical or prognosis implications.

#### Diagnostic criteria

The WHO CNS5 established the following essential diagnostic criteria for IMT, FET::CREB fusion-positive: 1/ primary intracranial location; 2/ variable morphological features including spindle cells, mucin-rich stroma, haemangioma-like vasculature, or epithelioid cells in a mucin-poor collagenous stroma; 3/ demonstration of a FET::CREB family fusion.

## ***CIC-***rearranged sarcoma

### Clinical characteristics

Most *CIC*-rearranged sarcomas of the CNS occur in supratentorial sites (85% of reported cases) [37–46] whereas spinal presentation accounts for 15% of reported cases [42, 46–48]. Presenting symptoms depend on the tumor’s location [37, 39, 44, 46]. There is a wide age range at presentation, from children to elderly adults (ranging from 0 to 71) [37–48]. However, there is a striking predilection for children and young adults (median age: 10 years), and 68% of cases are found in the pediatric age group [37–48]. There is no gender predisposition (sex ratio male/female of 1.2) [37–48]. Radiological data are scarce in the literature and limited to case reports [37–39, 43, 44, 47–49]. Tumors seem to manifest as a parenchymal hematoma (50% of reported cases) [37, 39, 44, 49] or as a solid and cystic mass (38% of reported cases) [38, 43, 47]. Like their soft tissue counterparts, most tumors follow an aggressive course with frequent recurrences (61% of reported cases), most commonly local [37–41, 43–48], resulting in death (38% of reported cases) [37–45, 47–49].

### Histopathology and cellular origin

These tumors are well-circumscribed from the brain parenchyma. They are mainly composed of diffuse sheets or lobules of undifferentiated round cells, epithelioid or even rhabdoid cells [37, 39, 41, 42, 44, 46–49]. Divergent differentiations (chondroid, glioneuronal with neuropil) have been described, which explain why some tumors were initially diagnosed as pleomorphic xanthoastrocytomas or gangliogliomas [38, 40, 44]. Similar glial/glioneuronal differentiation has not been reported to date in soft tissue counterparts with *CIC*-fusions [1–10]. A collagenous stroma or focal myxoid changes may be present in the tumor (Fig. 4E–H) [39, 43, 44, 46–49]. Necrosis is common and mitotic activity is brisk [37, 42, 44, 46, 47, 49]. When evaluated by immunohistochemistry, *CIC*-rearranged sarcomas may express, only focally or in a subset of tumor cells, markers of various differentiations (such as GFAP, CD56, synaptophysin, neurofilament, CKAE1/AE3, PS100, desmin, smooth muscle actin) [37, 38, 40, 44, 46–48]. A CD99 immunoexpression, which is frequently observed in their soft tissue counterparts, is focal or absent in CNS cases [37, 39, 46–48]. As in soft tissue [50, 51], WT1 and ETV4 are frequently positive and represent useful ancillary markers (Fig. 4H) [37, 39, 47, 49] and one recent study has shown its high sensitivity and specificity in the CNS compared to its other potential differential diagnoses [52]. Sarcomas with *CIC::NUTM1* fusions express NUT protein [42, 45]. The main differential diagnosis in the CNS is represented by the atypical teratoid and rhabdoid tumor which is easily ruled out by using immunohistochemistry staining for INI1 and BRG1. Although the cell of origin of *CIC-*rearranged sarcomas is still unknown, the fact that soft tissue and CNS tumors share the same DNA-methylation profiling is suggestive of a common mesenchymal origin [43].

### Molecular characteristics

Whereas *CIC::DUX4* fusion is encountered in 95% of *CIC-*rearranged sarcomas of the soft tissue, the molecular spectrum of CNS cases seems to be larger with different fusion partners: *CIC::LEUTX* (29%) [38, 40, 48], *CIC::NUTM1* (29%)[42, 45], *CIC::DUX4* (18%) [37, 46, 47], *ATXN1::DUX4* (12%) [43, 49], *ATXN1::NUTM1* (6%) [44], and a frameshift deletion of the *CIC* gene (6%) [45]. Whereas *CIC::LEUTX* and *CIC::NUTM1* have also been reported in a subset of *CIC-*rearranged sarcomas [42, 53], fusions implicating the *ATXN1* gene seem to be only encountered in CNS tumors. Whatever the type of fusion, a recent study has evidenced that *CIC-*rearranged sarcomas and *ATXN1*-rearranged sarcomas of the CNS share the same DNA-methylation signature and are not distinct from their soft tissue counterparts [43]. However, further series comparing CNS (particularly those showing glial/glioneuronal differentiation) and soft tissue tumors are needed to confirm these data.

### Diagnostic criteria

The WHO CNS5 listed the following essential diagnostic criteria: 1/ evidence of a *CIC* gene fusion; 2/ predominant round cell phenotype; 3/ mild nuclear pleomorphism; 4/ variable admixture of epithelioid and/or spindle cells; 4/variably myxoid stroma; 5/ variable CD99 and frequent ETV4 and WT1 expression. The DNA-methylation profile for CIC-rearranged sarcoma is a desirable diagnostic criterion.

## Primary intracranial sarcoma*, ****DICER1***-mutant

### Clinical characteristics

As its name suggests, the primary intracranial sarcoma, *DICER1*-mutant is almost exclusively encountered in supratentorial sites (92% of reported cases) [54–63]. However, exceptional infratentorial and spinal cases have been reported [54, 56, 58, 64]. Presenting symptoms depend on the tumor’s location. There is a wide age range at presentation, from children to elderly adults (ranging from 0 to 76) [54–64]. However, there is a striking predilection for children and young adults (median age: 6 years), and 87% of cases occur in the pediatric age group [54–64]. The gender distribution is almost equal (sex ratio female/male of 1.3) [54–64]. Radiological data are scarce in the literature and limited to case reports [57, 59–63]. The primary intracranial sarcoma, *DICER1*-mutant seems to present as a solid and cystic mass, with hemorrhage and leptomeningeal attachment, hypointensity on T1-weighted sequences, hyperintensity on T2-weighted images and a heterogeneous enhancement after gadolinium injection. Further series, including a radiological description of proven primary intracranial sarcoma, *DICER1*-mutant is needed to confirm these features. While the prognosis for patients with *DICER1*-mutant primary intracranial sarcoma remains to be determined, the literature data (36 cases) showed that 33% of patients presented local recurrences and that 86% of them are alive at the end of follow-up [54–57, 59–64]. A recent study has evidenced that a combination of surgery, chemotherapy, and radiotherapy seems to be beneficial in the treatment of this sarcoma subtype [56].

### Histopathology

*DICER1*-mutant primary intracranial sarcomas are mainly well-circumscribed tumors from the brain parenchyma and may present a leptomeningeal component. Histopathologically, they are pleomorphic or composed of spindle cells arranged in fascicules or a patternless growth [54, 55, 57–63, 65, 66]. A rhabdoid morphology or a myogenic differentiation (evidenced using desmin and/or myogenin markers) is frequently observed (Fig. 4I–L) [54, 55, 57–60, 62, 63, 65, 66]. The stroma may be myxoid and/or chondroid [54, 55, 57, 58, 62, 63, 65, 66]. Cytoplasmic eosinophilic globules PAS-positive are often present [54, 57, 59, 62, 63]. Using immunohistochemistry, they frequently express myogenic markers (desmin, smooth muscle actin, and occasionally myogenin), with variable intensity (focal or patchy) [54, 55, 57–64]. Because of the expression of S100 proteins, synaptophysin and neurofilament and the wide variety of differentiation (including lipomatous and pseudo-meissnerian components), a potential neural crest lineage has been suggested [63, 64]. A subset of reported cases has evidenced a p53 overexpression and a loss of ATRX expression [59, 61, 64]. GFAP, Olig2, and cytokeratins are not expressed [54, 55, 59–63]. *DICER1*-mutant primary intracranial sarcomas may present a complete or mosaic loss of H3K27me3 [54]. TLE1 immunopositivity has been suggested as a potential diagnostic surrogate [54], but the sensitivity and specificity of this biomarker needs to be studied in CNS tumors. Neoplasms from other organs showing a myogenic differentiation and harboring *DICER1* alterations have been reported in the literature (pleuropulmonary blastoma-like peritoneal sarcomas, *DICER1* renal sarcomas and rhabdomyosarcomas of the urogenital tract with *DICER1* mutations) [67–69], but their relationship (including epigenetic data) has to be elucidated before suggesting a potential unified terminology [70, 71].

### Molecular characteristics

A *DICER1* alteration is encountered in 98% of reported case [54–64] (only two cases proven by DNA-methylation profiling failed to reveal any mutation [56, 58]). *DICER1*-mutant primary intracranial sarcomas are characterized by a biallelic alteration of the *DICER1* gene combining a hotspot missense mutation on one allele and a truncating mutation (frameshift, nonsense, or splice-site) on the other [58]. Some tumors (5% of reported cases) harbor a single mutation accompanied by loss of heterozygosity eliminating the remaining wildtype allele [59, 63, 66]. A part of reported patients (26% of reported cases with available constitutional data) have a germline alteration of *DICER1*, as part of *DICER1* syndrome, and rare cases have been reported with a familial history of cancers (gynecological cancers) [55, 57, 58, 60, 64, 65, 72]. Primary intracranial sarcomas, *DICER1*-mutant present a high level of tumor mutational burden. Indeed, associated with *DICER1* alterations, recurrent mutations in the MAP-kinase pathway (mainly *KRAS, NF1* and *NRAS* genes) and *TP53* gene have been reported in 64% [54, 56–59, 63, 64, 72] and 60% of reported cases [54, 56–59, 62, 63, 72]. *DICER1*-mutant primary intracranial sarcoma harbors a distinct DNA methylation profile in the v12.5 version of the CNS tumor classification [58]. However, an epigenetic overlap with extracranial sarcomas harboring *DICER1* mutation remains undetermined.

### Diagnostic criteria

The current WHO classification has listed the following essential diagnostic criteria: 1/ primary intracranial sarcoma; 2/ pathogenic *DICER1* mutation (either germline or somatic). For unresolved lesions, the DNA-methylation profile for primary intracranial sarcoma, DICER1-mutant is mandatory.

## New insights for well-known tumors

### Rhabdomyosarcomas

Very few CNS cases in the literature have detailed histopathological and molecular characterizations. As reported in soft tissue, primary CNS alveolar rhabdomyosarcomas present fusions implicating *PAX3* and *FOXO1* genes (Fig. 4M–P) [73–76]. It has been evidenced that these fusions induce Olig2 expression by tumor cells, making this diagnosis challenging in the CNS (Fig. 4O) [75, 76]. Primary CNS alveolar rhabdomyosarcomas with proven *FOXO1* or *PAX3* fusions typically concern children and young adults with a pineal (3 cases) or a posterior fossa mass (1 case) [73–76]. In the pineal location, differential diagnoses of pediatric tumors with myogenic differentiation include atypical rhabdoid and teratoid tumors, medulloblastoma with divergent differentiations (particularly medullomyoblastoma), teratomas with a rhabdomyosarcomatous component, pineal anlage tumors and pineoblastomas with rhabdomyoblastic differentiation. These diagnoses may be obtained using clinical data, histopathology and immunohistochemistry. Data concerning the outcomes of cases proven molecularly are scarce and further reports are needed. The WHO CNS5 has listed the following essential diagnostic criteria: 1/ a malignant primitive tumour with at least focal immunohistochemical demonstration of skeletal muscle lineage; 2/ absence of non-rhabdomyosarcomatous components. The confirmation of a *FOXO1* gene fusion in diagnostically difficult cases is needed.

### Chordomas

Chordomas have reappeared as a specific taxonomic category in the current WHO classification [1]. This nosology clearly distinguishes four clinicopathological forms of chordomas: conventional, chondroid, dedifferentiated (which have historically represented the pejorative evolution of a classical chordoma following radiation therapy) and the poorly differentiated chordoma, *SMARCB1-*deficient. This last subtype mainly concerns children (86% of reported cases) with a median age of 7 (varying from 1 to 42 years-old) [77–93]. There is a slight female predominance (female to male ratio: 1.5) [77–93]. They are mainly located in the skull base (64% of reported cases) but may be encountered in the sacroccygeal region (27% of reported cases) or more rarely in the mobile vertebral column (9% of reported cases) [77–93]. Histopathologically, they are composed of cohesive sheets of epithelioid or spindle cells without chondromyxoid stroma and without physaliphorous cells (Fig. 4Q–R) [77–93]. A subset of cases having a classical morphology and INI1 loss have been reported and it remains uncertain if they represent the same clinicopathological type as the poorly differentiated form [77, 78, 93]. A diagnosis is made using the combination of a brachyury expression and the loss of INI1 protein immunoreactivity (Fig. 4S–T). It has been evidenced that poorly differentiated chordomas, *SMARCB1-*deficient are associated with a poor outcome with high rates of metastases (30% of reported cases) and death (43% of reported cases) [77–93]. To date, no distinct methylation class exists in the current version of the DKFZ classifier (v12.5). However, recent work that studied a cohort of CNS tumors with *SMARCB1* deficiency showed that poorly differentiated chordomas, *SMARCB1-*deficient constitute a different cluster [94]. The WHO CNS5 listed the following essential diagnostic criteria: 1/ a midline axial bone tumor; 2/ Lobules of cohesive and physaliphorous cells in a myxoid or chondroid matrix; 3/ Brachyury immunopositivity. In the case of epithelioid/solid forms, a loss of SMARCB1 (INI1) expression to confirm the diagnosis of poorly differentiated chordoma is mandatory.

## Emerging entities

### Dural angioleiomyomas

**Fig. 5:**
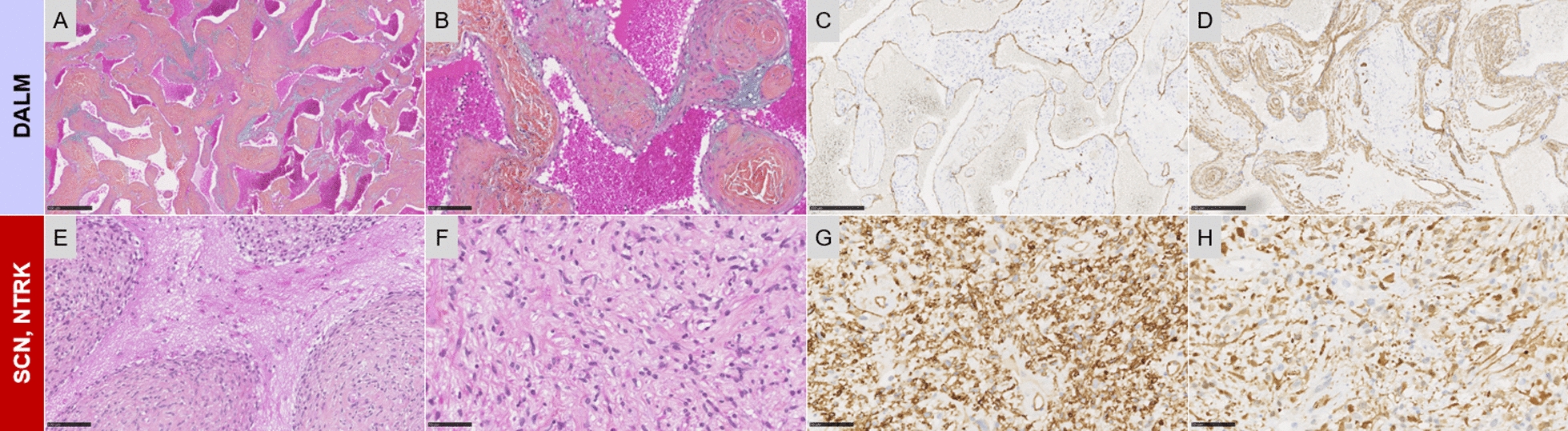
Histopathological findings of emerging entities. **A** Cavernous-type pattern composed of dilated vascular channels with variable thickening of the walls (HPS, magnification × 10) with **B** some perivascular concentric arrangement of myoid cells (HPS, magnification × 400). **C** The vascular cavities were lined by endothelial cells stained by CD34 (magnification × 400). **D** In the walls of vascular structure, the tumor cells were diffusely immunoreactive for h-caldesmon (magnification × 400). **E** Infiltration of the brain parenchyma (HPS, magnification × 400). **F** Spindle cell proliferation (HPS, magnification × 400). **G** Diffuse expression of CD34 (magnification × 400). **H** Strong expression of S100 (magnification × 400). Black scale bars represent 500 μm (**A**), 100 μm (**E**), and 50 μm (**B**–**D** and **F**–**H**). *DALM* dural angioleiomyoma, *HPS* hematoxylin phloxin saffron, *SCN* spindle cell neoplasm

Angioleiomyomas are well-known in the soft tissue and are classified within pericytic (perivascular) tumors in the WHO CNS5 (Fig. 5A–D) [95]. In the literature, dural presentations of a cavernous subtype of angioleiomyomas have been reported and a recent study performed a comprehensive clinicoradiologic and molecular characterization of a series [96]. Dural angioleiomyomas present clinical (affecting adults between the fourth and the sixth decades), and radiological similarities to soft tissue angioleiomyomas (such as hyperintensity on T2-weighted images and a strong enhancement after contrast injection) [96]. A subset presented the same p.Gly41Cys *GJA4* mutation, recently reported in other vascular lesions from different organs (liver, skin, orbit, soft tissue) [96–98]. Moreover, DNA methylation profiles indicate that dural angioleiomyomas grouped together and formed a distinct epigenetic group, separating them from the clusters of soft tissue angioleiomyomas, other vascular tumors, inflammatory myofibroblastic tumors and meningiomas. The extensive literature review identified several cases similar to these lesions, with a wide variety of denominations (mainly named as cavernous hemangiomas and veinous hemangiomas). Because of its dural location and distinct methylome profile, a potential terminology to designate this benign tumor could be “dural angioleiomyoma”. Further studies are needed to confirm its inclusion in a future version of the WHO classification of CNS tumors.

### Spindle cell neoplasms, ***NTRK-***rearranged

*NTRK* gene fusions have been described in a wide variety of CNS and soft tissue tumors, including the provisional tumor type “spindle cell neoplasm, *NTRK*-rearranged” (SCN-NTRK), added to the 2020 WHO Classification of Soft Tissue Tumors. Because of histopathological and molecular overlaps with other soft tissue entities, controversy remains concerning the lineage and terminology of SCN-NTRK. Rare CNS primary presentations of SCN-NTRK have been reported in the literature [99–101]. A recent series including soft tissue and CNS cases revealed similar histopathological, immunophenotypical, and molecular (spindle cell tumors with coexpression of CD34 and S100 and a *CDKN2A* homozygous deletion) features and formed a unique and new methylation cluster (Fig. 5E–H) [101]. These tumors are predominately found in children and young adults [99–101]. While a recent study evidenced that SCN-NTRK share similar features in all locations, SCN-NTRK are probably underdiagnosed, and further cases of CNS SCN-NTRK are needed to confirm or not their place in the next WHO Classification of CNS tumors.

## Conclusion

Several mesenchymal non-meningothelial tumors have now been defined by specific molecular alterations, with some being exclusive to the CNS. In this respect, the WHO CNS5 represents an extension of the changes first introduced by the former edition. The increased precision in decipherment was achieved by novel genetic and DNA-methylation diagnostic technologies. The utility of this last methodology is particularly interesting for mesenchymal tumors because of the existence of two different classifiers (one for brain tumors and one for soft tissue tumors). This increased complexity reflects our current understanding of biological features of CNS tumors. However, great effort is now necessary to 1/ compare them to their extra-CNS counterparts; 2/ to more precisely characterize the clinical and radiological aspects and outcomes of these new tumor types, and eventually 3/ to determine a grading (no grade is currently associated with these novel tumor types) to adapt therapeutic approaches in the future.
